# Abundance considerations for modeling yield of rapeseed at the flowering stage

**DOI:** 10.3389/fpls.2023.1188216

**Published:** 2023-07-28

**Authors:** Yuanjin Li, Ningge Yuan, Shanjun Luo, Kaili Yang, Shenghui Fang, Yi Peng, Yan Gong

**Affiliations:** School of Remote Sensing and Information Engineering, Wuhan University, Wuhan, China

**Keywords:** rapeseed, yield estimation, spectral mixture analysis, optical remote sensing, vegetation index

## Abstract

**Introduction:**

To stabilize the edible oil market, it is necessary to determine the oil yield in advance, so the accurate and fast technology of estimating rapeseed yield is of great significance in agricultural production activities. Due to the long flowering time of rapeseed and the characteristics of petal color that are obviously different from other crops, the flowering period can be carefully considered in crop classification and yield estimation.

**Methods:**

A field experiment was conducted to obtain the unmanned aerial vehicle (UAV) multispectral images. Field measurements consisted of the reflectance of flowers, leaves, and soils at the flowering stage and rapeseed yield at physiological maturity. Moreover, GF-1 and Sentinel-2 satellite images were collected to compare the applicability of yield estimation methods. The abundance of different organs of rapeseed was extracted by the spectral mixture analysis (SMA) technology, which was multiplied by vegetation indices (VIs) respectively to estimate the yield.

**Results:**

For the UAV-scale, the product of VIs and leaf abundance (AbdLF) was closely related to rapeseed yield, which was better than the VIs models for yield estimation, with the coefficient of determination (R2) above 0.78. The yield estimation models of the product of normalized difference yellowness index (NDYI), enhanced vegetation index (EVI) and AbdLF had the highest accuracy, with the coefficients of variation (CVs) below 10%. For the satellite scale, most of the estimation models of the product of VIs and rapeseed AbdLF were also improved compared with the VIs models. The yield estimation models of the product of AbdLF and renormalized difference VI (RDVI) and EVI (RDVI×AbdLF and EVI×AbdLF) had the steady improvement, with CVs below 13.1%. Furthermore, the yield estimation models of the product of AbdLF and normalized difference VI (NDVI), visible atmospherically resistant index (VARI), RDVI, and EVI had consistent performance at both UAV and satellite scales.

**Discussion:**

The results showed that considering SMA could improve the limitation of using only VIs to retrieve rapeseed yield at the flowering stage. Our results indicate that the abundance of rapeseed leaves can be a potential indicator of yield prediction during the flowering stage.

## Introduction

Remote sensing plays an important role in agricultural applications, and various remotely obtained information is urgently needed by decision-makers (Atzberger, 2013). Remote sensing with unmanned aerial vehicles (UAVs) offering unprecedented spectral, spatial, and temporal resolution but also providing detailed vegetation height data and multi-angular observations is a game-changer in precision agriculture (Maes and Steppe, 2019). Precision agriculture can be broadly meant as an agricultural system in which the management practice is performed at a suitable place, with the appropriate intensity, and at the right time. Precision agriculture uses intensive data and information collection and processing in time and space to make more efficient use of farm inputs, leading to improved crop production (Mulla, 2013). The framework of deep neural network (DNN) by UAV-based multimodal data fusion using RGB, multispectral, and thermal sensors was used in soybean grain yield estimation, which improved yield prediction accuracy and adaptability of spatial variations. However, the model requires too many parameters and is not easy to implement (Maimaitijiang et al., 2020). A random forest model based on a dual-camera high-throughput phenotyping (HTP) platform was used to measure crop geometric features and obtain high correlations with final yield in breeding populations, but the cost of a stable carrier and a high-quality sensor system became the current limitations of HTP (Yu et al., 2016). It is demonstrated that both multispectral and digital sensors mounted on the UAV are reliable platforms for rice growth and grain yield estimation. Moreover, for rice grain, the best period, the booting stage, and optimal vegetation indices (VIs) for yield prediction were determined (Zhou et al., 2017). However, for different crops, this conclusion remains to be verified. UAV-based VIs and abundance information obtained from spectral mixture analysis (SMA) were integrated to improve the estimation accuracy of rice yield at the heading stage (Duan et al., 2019). UAV remote sensing can establish a robust model according to different crops, but the transferability of the model needs further research.

Compared with UAV remote sensing, satellite remote sensing can reflect the spatial and spectral information of ground objects on a larger scale and is often applied in estimating large-scale crop productivity (Toth and Jóźków, 2016), which has critical value for scientific and societal benefits (Lobell et al., 2009). In addition, large-scale crop yield estimation often relies on satellite remote sensing (Guan et al., 2017). Traditional approaches have primarily used visible and near-infrared (NIR) remote sensing data. Many studies directly used the satellite remote sensing-based VI models that provided a general indicator of vegetation features to estimate crop yield (Son et al., 2014; Munghemezulu et al., 2017; Nagy et al., 2018; García-Martínez et al., 2020). The normalized difference vegetation index (NDVI) is one of the first VIs and has been widely used for vegetation monitoring (Tucker, 1979). After that, various VIs were derived for different crops and environments. Near real-timely U.S. corn yields based on time-series MODIS data with wide dynamic range vegetation index (WDRVI) and bias correction algorithm were predicted (Sakamoto et al., 2014), but because of the coarse spatial resolution (250 m) and the mixed-pixel effect, the proposed method would have limited applicability to other regions of the globe. A raw imagery-based deep learning approach for field-scale yield prediction with in-season multitemporal imagery was developed (Sagan et al., 2021). The approach could explain nearly 90% variance in field-scale yield, but it contained hundreds of spectral, spatial, textural, and temporal features and was too complicated. Spatiotemporal fusion of Landsat-8 and MODIS data to derive phenology, biomass, and yield estimation for corn and soybean was used (Liao et al., 2019), which solved the problem of interference of cloud cover and rather long revisiting cycles of high-resolution satellite sensors to some extent. However, this approach needed images from multiple periods and had a time-consuming pixel-based optimization procedure. Several VIs and machine learning (ML) approaches were compared to map within-field wheat grain yield by combining harvester data and EOS Sentinel-2 multispectral bands (Segarra et al., 2022). However, the methods in most papers mainly estimated crop yield by combining multiple time series data and ML approaches. Although there was also reliable estimation accuracy, the data acquisition cycle was long and often covered the entire crop growth period. In addition, these methods rarely optimize for the characteristics of the crops themselves or reduce the complexity of the methods. Moreover, for large-scale grain yield estimation, mixed-pixel effects from image resolution may negatively impact grain yield estimation.

We aim to utilize the growth characteristics of crops. Based on accurate identification, we used the main characteristics that affect yield to estimate yield. On the one hand, combined with the flowering characteristics of rapeseed, we used UAV multispectral images to develop VI-improved models of different organs based on the SMA for yield estimation. On the other hand, we used GF-1 wide-field view (WFV) images to extract the rapeseed planting area of the Jianghan Plain in Hubei Province at the flowering stage and transferred the approach from the UAV scale to the satellite scale to further demonstrate the approach.

This study focused on the following questions: i) How do we explore a simpler and easier yield estimation model for the flowering characteristics of rapeseed crops? ii) Is the approach consistent across scales? iii) Which VI is more suitable for the rapeseed yield estimation combined with the flowering characteristics? Combining the above problems, this study developed a simple and feasible yield estimation model according to the flowering characteristics of rapeseed crops, which can estimate yield only based on the flowering period of rapeseed and provide data support for economic decisions, ensuring farmers’ income and food security.

## Materials and methods

### Experimental design

**Figure 1:**
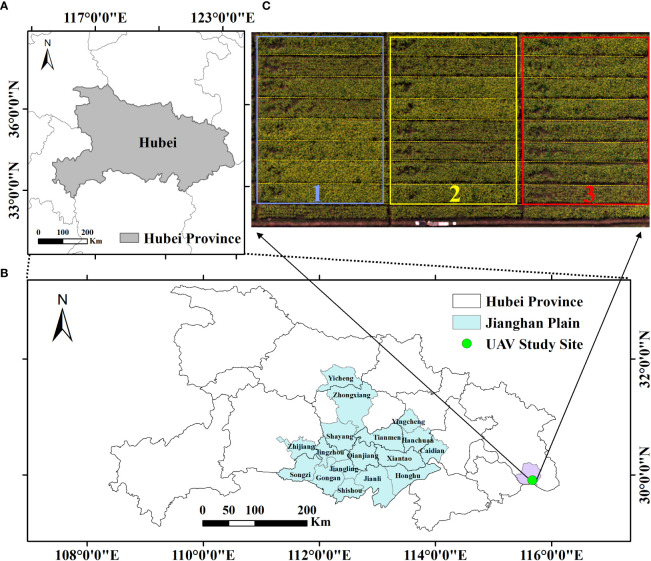
Study site. **(A)** General location of the study area. **(B)** Satellite study site. **(C)** Details of the UAV experimental design. Three rectangular boxed (blue, yellow and red) areas represent triplicate experiments. UAV, unmanned aerial vehicle.

There were two study sites in this study, including the UAV field experiment and the satellite large-scale experiment (**Figure 1A**).

The UAV rapeseed experiment was conducted from October 2014 to May 2015 at Rapeseed Experiment and Research Base, Wuxue, Hubei, China. We studied 24 rapeseed plots, with the size of 15 m × 2 m, and all planted with the same hybrid of rapeseed (Huayouza No. 9) (Ma et al., 2014). The field management for these plots was similar except that different amounts of nitrogen fertilizer were applied. Eight nitrogen (N) rates (0, 45, 90, 135, 180, 225, 270, and 360 kg/ha) were utilized based on the available research (Ren et al., 2016), and each rate was repeated on three randomly distributed plots (**Figure 1C**).

Another study site was in the Jianghan Plain, which was located in the middle reaches of the Yangtze River, in the central and southern parts of Hubei Province. The Jianghan Plain belongs to the northern subtropical humid monsoon climate, with distinct four seasons, sufficient light energy resources, very rich heat resources, a long frost-free period, abundant rainfall, and the same period of rain and heat, which is very suitable for rapeseed cultivation (**Figure 1B**).

### Data acquisition

#### UAV data acquisition

The UAV flight was carried out on 2 March 2015 between 10:00 and 13:00 local time when changes in solar zenith angle were minimal at a 50-m altitude to collect the centimeter-level images with a 2.74-cm spatial resolution, and the weather was clear with low cloud cover observed. The Mini-MCA system (Mini-MCA 6, Tetracam Inc., Chatsworth, CA, USA) was mounted on a UAV (S1000, SZ DJI Technology Co., Ltd., Shenzhen, China) to obtain images of the studied area. Mini-MCA consists of six individual miniature digital cameras [central bands of 490@10 (B), 550@10 (G), 670@10 (R), 720@10 (RE), 800@10 (NIR1), and 900@20 nm (NIR2)]. These bands were selected since they were commonly used for estimating vegetation photosynthesis-related parameters (Behrens et al., 2006; Ray et al., 2010; Kira et al., 2015).

Prior to the flight, three calibration ground targets with the reflectance of 0.06/0.24/0.48 were laid on the study area for UAV radiometric calibration (more details can be found at https://www.tetracam.com/Products_Ground_Calibration_Panels.htm). When flying, the cameras were fixed on the gimbal to ensure that the lenses were always on the horizontal plane, which could minimize the change of ground reflectance caused by the observation angles. Each time the cameras were exposed, they could obtain six 8-bit RAW format images at the same time.

This study used an empirical linear model (ELM) to convert UAV image digital number (DN) values to ground reflectance (Dwyer et al., 1995; Laliberte et al., 2011). ELM assumes that there is a linear relationship between the DN values and the ground reflectance, and the canopy reflectance can be calculated by the following formula (Dwyer et al., 1995; Baugh and Groeneveld, 2008):


(1) ρ(λ)=gains(λ)×DN(λ)+bias(λ)

(λ= 490, 550, 670, 720, 800, and 900 nm),

where DN(λ) is the digital number of a pixel at the band with the wavelength λ; gains(λ) and bias(λ) are gains and bias of the camera at wavelength λ , respectively. Gains and bias can be calculated from DN values by three calibration targets for each band. Within each of the 24 plots, a rectangular region of interest (ROI) was defined for each plot by avoiding plot boundaries, and the mean reflectance within the region was treated as the plot-level reflectance of the plot. Plot-level VIs were then retrieved from plot-level canopy reflectance (**Table 1**).

**Table 1:** Vegetation indices selected in this study.

Vegetation indices	Abbreviation	Formula	References
Normalized difference vegetation index	NDVI	$(\rho_{800nm}-\rho_{670nm})/\left(\rho_{800nm}+\rho_{670nm}\right)$	(Rouse et al., 1974)
Green chlorophyll index	CI_green_	$\rho_{800nm}/\rho_{550nm}-1$	(Gitelson, 2005)
Visible atmospherically resistant index	VARI	$(\rho_{550nm}-\rho_{670nm})/\left(\rho_{550nm}+\rho_{670nm}\right)$	(Gitelson et al., 2002)
Ratio vegetation index	RVI	$\rho_{800nm}/\rho_{670nm}$	(Jordan, 1969)
Difference vegetation index	DVI	$\rho_{800nm}-\rho_{670nm}$	(Richardson and Wiegand, 1977)
Renormalized difference vegetation index	RDVI	$(\rho_{800nm}-\rho_{670nm})/\sqrt{\left(\rho_{800nm}+\rho_{670nm}\right)}$	(Roujean and Breon, 1995)
Enhanced vegetation index	EVI	$2.5(\rho_{800nm}-\rho_{670nm})/\left(\rho_{800nm}+6\rho_{670nm}-7.5\rho_{490nm}+1\right)$	(Liu and Huete, 1995)
Triangular vegetation index	TVI	$0.5\left[120(\rho_{800nm}-\rho_{550nm}\right)-200\left(\left(\rho_{670nm}-\rho_{550nm}\right)\right]$	(Broge and Leblanc, 2001)
Normalized difference yellowness index	NDYI	$(\rho_{550nm}-\rho_{490nm})/\left(\rho_{550nm}+\rho_{490nm}\right)$	(Sulik and Long, 2015)

ρ stands for reflectance.

#### Satellite data acquisition

The satellite dataset used in this study was composed of five GF-1 WFV images and 19 Sentinel-2 images covering the Jianghan Plain. A summary of information regarding the sensor, resolution, acquisition date, number, and phenology stage for these images is listed in **Table 2**. The GF-1 WFV images were downloaded from the China Center for Resources Satellite Date and Application (CRESDA), which had four bands (blue, green, red, and NIR). Sentinel-2 data include all available Sentinel-2A and Sentinel-2B Multi Spectral Instrument (MSI) images from the European Space Agency (ESA). The detailed information could be browsed on the website of CRESDA and ESA.

**Table 2:** A summary of information on satellite images.

Sensor	Resolution	Acquisition date	Number	Phenology stage
GF-1 WFV	16 m	17 March 2014	2	Flowering stage
		26 March 2014	2	
		29 March 2014	1	
Sentinel-2A/B	10 m	9 March 2018	13	
		3 April 2018	4	
		8 April 2018	2	

The preprocessing of GF-1 WFV images includes geometric correction, radiometric calibration, and atmospheric correction. A geometric correction was conducted with the assistance of ASTER GDEM V2 data (Zhang et al., 2015). For each image, it was processed using the Environment for Visualizing Images (ENVI) 5.3 software (Harris Geospatial Solutions, Inc., Broomfield, CO, USA) with the updating calibration parameters published in CRESDA, obtained by a large number of calibration experiments in Chinese calibration fields. In this study, an atmospheric correction was performed using the FLASH model in ENVI. The Sentinel-2 data were preprocessed by Sen2Cor supplied by ESA from https://step.esa.int/main/snap-supported-plugins/sen2cor/sen2cor-v2-10/. The rapeseed planting area could be extracted easily by using a colorimetric transformation and spectral feature-based oilseed rape extraction algorithm (CSRA) due to the obvious color characteristics of rapeseed during flowering (Wang et al., 2018).

#### Rapeseed yield determination

The 24 rapeseed plots at Wuxue City were harvested on 5 May 2015. In each plot, half of the rapeseed plants (approximately 15 m^2^) were all cut for yield determination, and they were exposed to the sun for 10 days for seed threshed. The seeds were then cleaned and put into an oven at 60°C until their weight did not change. After that, the plot yield per unit area (kg/ha) was converted by total weight and ground area.

#### Ancillary data

The statistical planting areas and yields of rapeseed in Hubei Province in 2014 and 2018 at county levels were used to validate the yield models. The statistical data were downloaded from the Hubei Statistical Bureau (https://tjj.hubei.gov.cn/tjsj/sjkscx/tjnj/gsztj/whs/).

### Spectral mixture analysis

**Figure 2:**
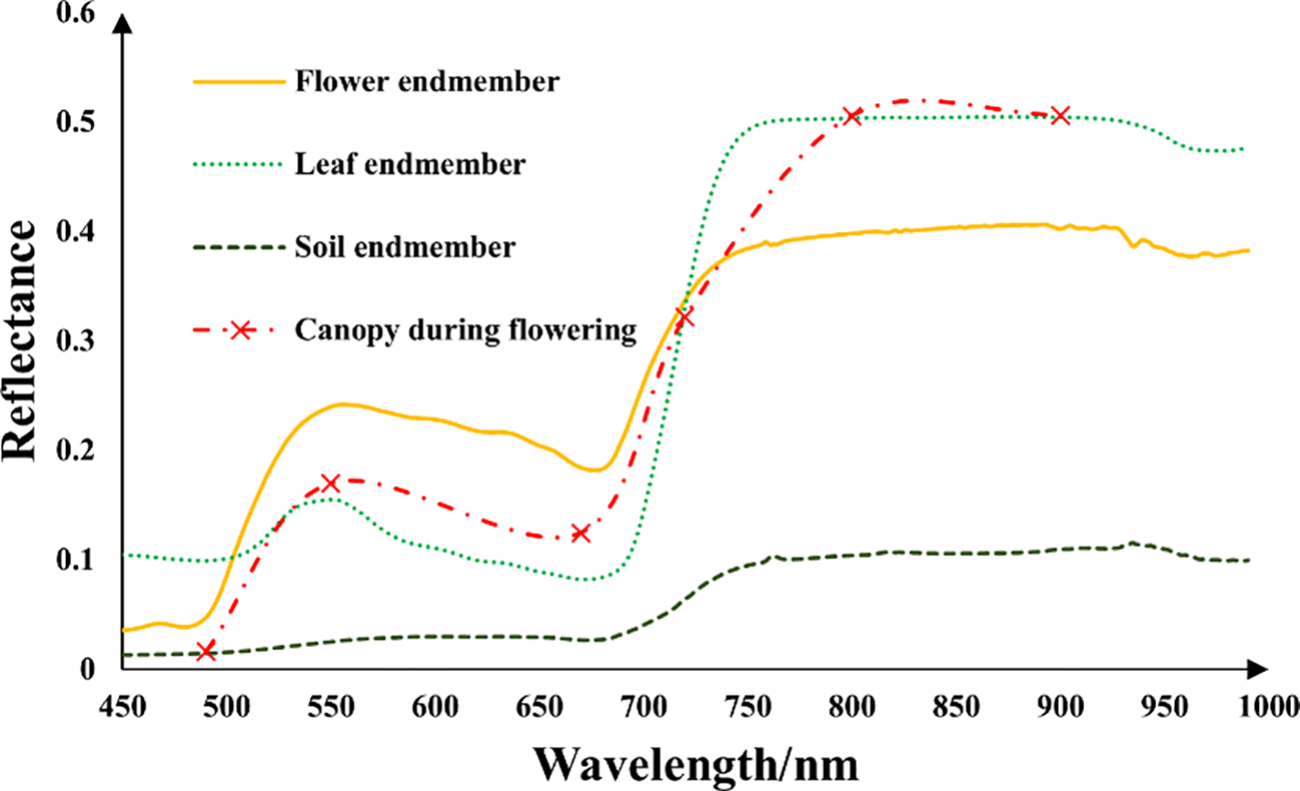
Pure spectral reflectance from 450 to 1,000 nm of flower endmember ( $\rho_{FL})$ , leaf endmember ( $\rho_{LF})$ , and soil endmember ( $\rho_{SL})$ in the studied rapeseed plots measured by Analytical Spectral Devices (ASD) and rapeseed canopy reflectance during flowering from Mini-MCA.


A pixel often contains multiple spectral members. This study needs to analyze the effects of different spectral components in rapeseed fields on rapeseed yield. Therefore, three endmembers were considered in this study: 1) flower (FL), 2) leaf (LF), and 3) soil (SL). They were the dominant components visible in our studied scene. Samples of each endmember were collected from the 24 plots, and their spectra were immediately measured *in situ* by a hyperspectral radiometer (Analytical Spectral Devices Inc., Boulder, CO, USA). This radiometer was equipped with a 25° field-of-view optical fiber that obtained sample reflectance in the range of 350–2,500 nm at a spectral resolution of approximately 1 nm. The measurements were conducted in all plots (at least six sampling areas per plot). The leaf spectra were taken by the radiometer with a self-illuminated leaf clip. Since the rapeseed flower is small and narrow, the sample flowers were gathered together on a black background and arranged to fully cover the sensor’s view field to make sure that the radiometer collected the pure spectra of flowers. The averaged spectra were used as endmember spectra of flowers. In this way, the reference endmember reflectance of three components was obtained (**Figure 2**):

(2) $$\rho \left(\lambda \right)=\sum_{i=1}^{N}Abd_{i}\rho_{i}\left(\lambda \right);\;0\leq Abd_{i}\leq 1\;\text{and}\;\sum_{i=1}^{N}Abd_{i}=1$$

and $\rho_{SL}$ .

For SMA, the linear mixing spectral model was used in this study to estimate the fractional abundance of each spectral endmember (Singer et al., 1979). It is assumed that the pixels in the acquired image can be represented as a linear mixture of a few dominant spectral endmembers. For a given pixel at the wavelength λ , the pixel reflectance ρ(λ)  can be approximated as follows:


, (2) $$\rho \left(\lambda \right)=\sum_{i=1}^{N}Abd_{i}\rho_{i}\left(\lambda \right);\;0\leq Abd_{i}\leq 1\;\text{and}\;\sum_{i=1}^{N}Abd_{i}=1$$

where *N* is the number of selected endmembers, $Abd_{i}$ is the fractional abundance of endmember i , and $\rho_{i}\left(\lambda \right)$ is the reference reflectance of endmember i at band λ . According to Equation 2, the abundance of the selected three endmembers for each pixel can be retrieved from the six-band UAV images and GF-1 WFV images (Heinz et al., 1999; Pu et al., 2015; Pan et al., 2017):


(3) $$\left[\begin{array}{c} \rho \left(\lambda 1\right)\\ \begin{array}{c} \rho \left(\lambda 2\right)\\ \begin{array}{c} \rho \left(\lambda 3\right)\\ \begin{array}{c} \rho \left(\lambda 4\right)\\ \rho \left(\lambda 5\right)\\ \rho \left(\lambda 6\right) \end{array} \end{array} \end{array} \end{array}\right]=\left[\begin{array}{l} \rho_{FL}(\lambda 1)\rho_{LF}(\lambda 1)\rho_{SL}(\lambda 1)\\ \rho_{FL}(\lambda 2)\rho_{LF}(\lambda 2)\rho_{SL}(\lambda 2)\\ \rho_{FL}(\lambda 3)\rho_{LF}(\lambda 3)\rho_{SL}(\lambda 3)\\ \rho_{FL}(\lambda 4)\rho_{LF}(\lambda 4)\rho_{SL}(\lambda 4)\\ \rho_{FL}(\lambda 5)\rho_{LF}(\lambda 5)\rho_{SL}(\lambda 5)\\ \rho_{FL}(\lambda 6)\rho_{LF}(\lambda 6)\rho_{SL}(\lambda 6) \end{array}\right]\left[\begin{array}{c} Abd_{FL}\\ Abd_{LF}\\ Abd_{SL} \end{array}\right]$$

(4) $$\left[\begin{array}{c} \rho \left(\lambda 1\right)\\ \begin{array}{c} \rho \left(\lambda 2\right)\\ \begin{array}{c} \rho \left(\lambda 3\right)\\ \rho \left(\lambda 4\right) \end{array} \end{array} \end{array}\right]=\left[\begin{array}{l} \rho_{FL}\left(\lambda 1\right)\rho_{LF}\left(\lambda 1\right)\;\rho_{SL}\left(\lambda 1\right)\\ \rho_{FL}\left(\lambda 2\right)\rho_{LF}\left(\lambda 2\right)\;\rho_{SL}\left(\lambda 2\right)\\ \rho_{FL}\left(\lambda 3\right)\rho_{LF}\left(\lambda 3\right)\;\rho_{SL}\left(\lambda 3\right)\\ \rho_{FL}\left(\lambda 4\right)\rho_{LF}\left(\lambda 4\right)\;\rho_{SL}\left(\lambda 4\right) \end{array}\right]\left[\begin{array}{c} Abd_{FL}\\ Abd_{LF}\\ Abd_{SL} \end{array}\right].$$

Formulas 3 and 4 are the calculation formulas for the endmember abundance of UAV images and satellite images, respectively, where ρ(λi) is the surface reflectance of the given pixel at $band_{i}$ ( UAV: i  = 1, 2, …, 6; GF-1: i  = 1,2, …, 4; Sentinel-2: i  = 2, 3, 4, 8; this paper focused only on the red, green, blue, and near-infrared bands in order to be consistent with GF-1). $\rho_{FL}\left(\lambda_{i}\right),\;\rho_{LF}\left(\lambda_{i}\right),\;$ and $\rho_{SL}\left(\lambda_{i}\right)$ are the endmember reflectance at $band_{i}$ for flowers, leaves, and soil at the study site, respectively. The $Abd_{FL}$ , $Abd_{LF},$ and $Abd_{SL}$ are the abundance of flower, leaf, and soil, respectively, referring to the fraction of the given component within a pixel.

### Yield estimation based on combination of vegetation indices and abundance

Leaves are crucial organs for photosynthesis and yield accumulation of rapeseed, so it is worth trying to estimate yield by studying the spectral characteristics of rapeseed. In yield estimation using VIs, the mixed effect of pixels in different scales is often a significant factor affecting the accuracy of the model. The flowers and leaves of rapeseed are important functional organs, which were separated from pixels and studied separately. Therefore, this study extracted the abundance of flowers and leaves through SMA to separately study the role of different organ spectra in yield analysis while removing the effect of soil spectra, combined with the vegetation index model, to jointly estimate yield. In this study, 24 plots in Wuxue City and 15 counties in the Jianghan Plain were used to establish three linear models with yield: i) yield versus VI, ii) yield versus VI × Abd_FL_, and iii) yield versus VI × Abd_LF_. Coefficients of determination (R^2^), root mean square error (RMSE), and coefficient of variation (CV) were analyzed and compared.

### Algorithm establishment using leave−one−out cross−validation

Due to the limited sample size in this study, the leave-one-out cross-validation approach was used to validate the results (Fielding and Bell, 1997). The R^2^, RMSE, and CV were selected to value the performance of the models. The correlated algorithms with accuracy can be produced as follows:


, (5) $$R^{2}=\frac{\sum_{i=1}^{K}R_{i}^{2}}{K}$$

(6) $$RMSE=\sqrt{\frac{\sum_{i=1}^{K}E_{i}^{2}}{K}},$$

(7) $$CV=\frac{1}{K}\sum_{i=1}^{N}CV_{i},$$

where *K* is the number of samples. For each time *i*, *K* − 1 samples were used iteratively as training data.

## Results

### Extraction of rapeseed based on GF-1 WFV images in Jianghan Plain

**Figure 3:**
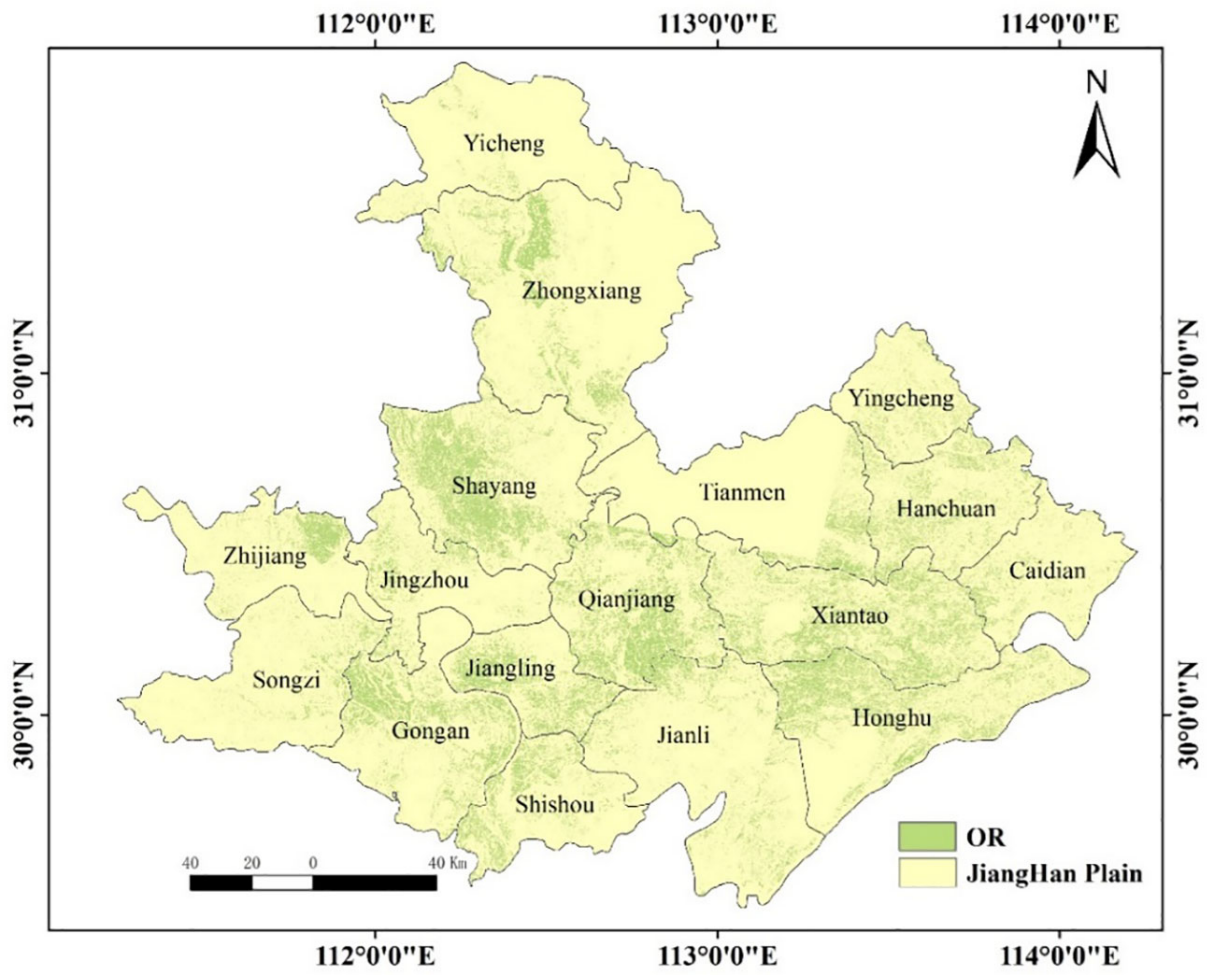
Distribution map of rapeseed in Jianghan Plain in 2014. OR, oilseed rape.

**Figure 4:**
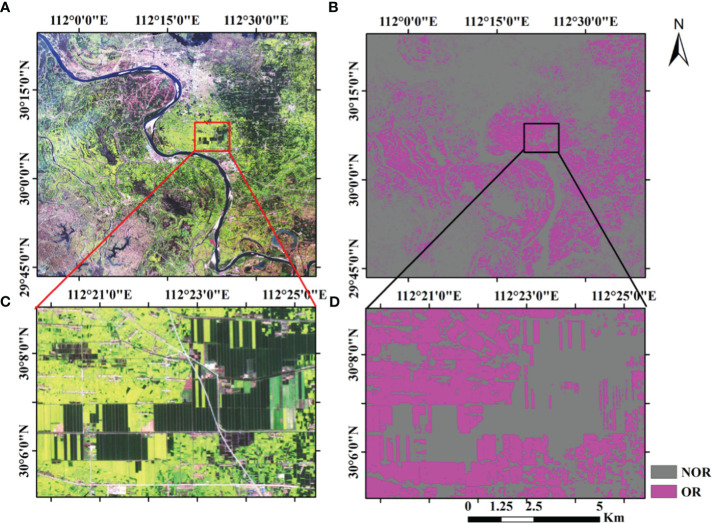
Rapeseed planting area extraction renderings. **(A)** The GF-1 image of rapeseed during flowering. **(B)** The mask image of rapeseed was obtained using CSRA. **(C, D)** The details of the rapeseed before and after extraction. NOR, no oilseed rape; OR, oilseed rape.

The following takes the extraction of the rape planting area from the GF-1 multispectral image as an example. **Figure 3** shows the distribution of flowering rapeseed in the Jianghan Plain in 2014, which was estimated by CSRA (Wang et al., 2018). Due to cloud cover, there was only a small distribution of rapeseed in Yicheng, Tianmen, and Jingzhou counties among the GF-1 WFV images available during flowering. As could be seen from **Figure 3**, rapeseed was mainly distributed in Shayang, Zhongxiang, Qianjiang, Xiantao, Jiangling, Gongan, Honghu, Hanchuan, and other regions. The GF-1 image of rapeseed during flowering is shown in **Figure 4A**, and the mask image of rapeseed was obtained using CSRA (**Figure 4B**). The flowering rapeseed showed bright yellow in the image, which was different from other vegetation. The details of the rapeseed are shown in **Figures 4C, D**. Most of the rapeseed was extracted very accurately, but there was a fuzzy phenomenon in the boundary of the features. The confusion matrix could analyze the spatial distribution of classification (**Table 3**). Classification accuracy was well assessed using visual interpretation methods. The overall accuracy was 88.40%. The user accuracy was relatively low (83.62%). Producer accuracy indicated that the CSRA-derived OR maps using GF-1 WFV images could obtain high spatial consistency. Rapeseed from Sentinel-2 data was extracted just like GF-1 WFV images do. Therefore, the extracted rapeseed map could be used as data for subsequent yield analysis.

**Table 3:** Confusion matrix of local accuracy assessment by visual interpretation.

Class	NOR	OR	UA (%)
NOR	27,418	2,226	92.49
OR	4,163	21,249	83.62
PA (%)	86.82	90.52	–
OA (%)	88.40	–	–
Kappa	0.77	–	–

PA, producer accuracy; UA, user accuracy; OA, overall accuracy; NOR, no oilseed rape; OR, oilseed rape.

### Relationship of VI versus yield in rapeseed

**Figure 5:**
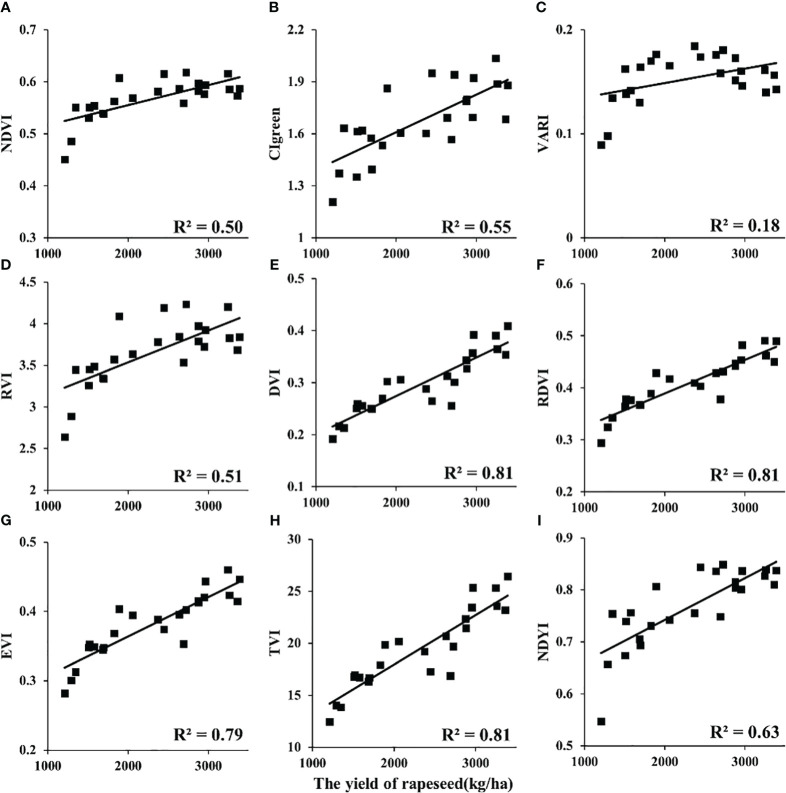
Relationship between VIs and rapeseed yield at the UAV scale. VIs, vegetation indices; UAV, unmanned aerial vehicle. (**A**: NDVI, **B**: CIgreen, **C**: VARI, **D**: RVI, **E**: DVI, **F**: RDVI, **G**: EVI, **H**: TVI, I: NDYI).

**Figure 6:**
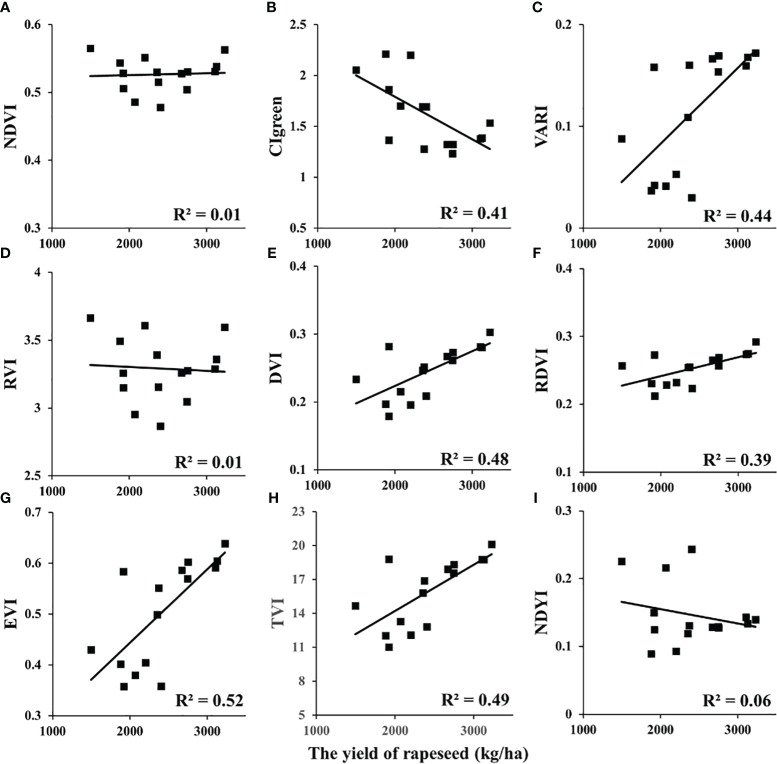
Relationship between VIs and rapeseed yield at the satellite scale: GF-1 in 2014. VIs, vegetation indices. (**A**: NDVI, **B**: CIgreen, **C**: VARI, **D**: RVI, **E**: DVI, **F**: RDVI, **G**: EVI, **H**: TVI, **I**: NDYI).

**Figure 7:**
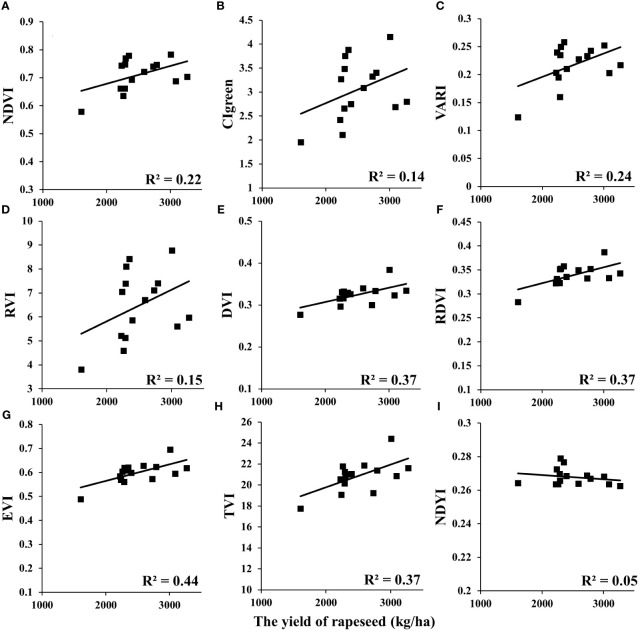
Relationship between VIs and rapeseed yield at the satellite scale: Sentinel-2A/B in 2018. VIs, vegetation indices. (**A**: NDVI, **B**: CIgreen, **C**: VARI, **D**: RVI, **E**: DVI, **F**: RDVI, **G**: EVI, **H**: TVI, **I**: NDYI).

In this study, the relationships between VIs and yield at different scales (UAV and satellite) were analyzed (**Table 4**). Among these VIs, DVI, renormalized difference VI (RDVI), and triangular vegetation index (TVI) showed significant correlations with yield (R^2^ > 0.8) of rapeseed at the UAV scale. Enhanced vegetation index (EVI) and normalized difference yellowness index (NDYI) showed strong correlations (R^2^ > 0.6). The remaining VIs showed a medium correlation (R^2^< 0.52). In addition, the NDVI, visible atmospherically resistant index (VARI), and RVI versus yield appeared non-linear in **Figure 5**. For GF-1 WFV images in 2014 (**Figure 6**), the EVI, CIgreen, VARI, DVI, RDVI, and TVI displayed medium correlation with the statistical yield by the National Statistical Bureau. Unlike the UAV scale, NDVI, RVI, and NDYI from GF-1 showed no correlation with statistical yield (R^2^< 0.1) (**Figures 6A, D and I**). Even more differently, CIgreen showed a negative correlation with yield in **Figure 6B**. For Sentinel-2 data in 2018, the EVI, DVI, RDVI, and TVI displayed a medium correlation with the statistical yield by National Statistical Bureau. Others showed weak or no correlation in **Figure 7**.

**Table 4:** Coefficients of determination (R^2^) between VIs and yield in rapeseed.

	NDVI	CI_green_	VARI	RVI	DVI	RDVI	EVI	TVI	NDYI
UAV	0.50	0.55	0.18	0.51	0.81*	0.81	0.79*	0.81*	0.63
GF-1	0.01	0.41	0.44	0.01	0.48*	0.39	0.52*	0.49*	0.06
Sentinel-2A/B	0.22	0.14	0.24	0.15	0.37*	0.37	0.44*	0.37*	0.05

Among them, the part marked with * and bold is the first three parameters with good performance in different scales.

VIs, vegetation indices; NDVI, normalized difference vegetation index; CI_green_, green chlorophyll index; VARI, visible atmospherically resistant index; RVI, ratio vegetation index; DVI, difference vegetation index; RDVI, renormalized difference vegetation index; EVI, enhanced vegetation index; TVI, triangular vegetation index; NDYI, normalized difference yellowness index; UAV, unmanned aerial vehicle.

### Variation of abundance and yield with nitrogen application

**Figure 8:**
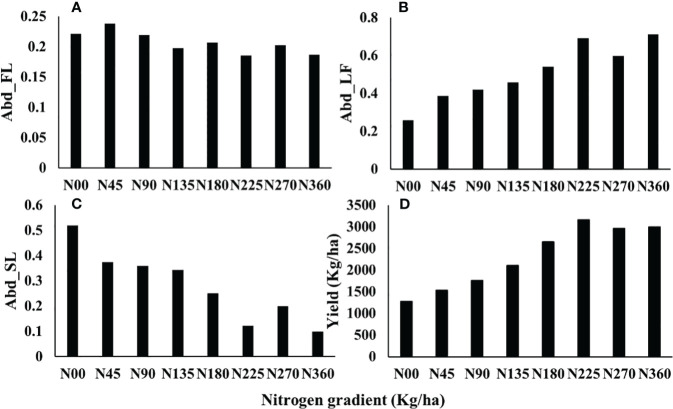
Variation of abundance and yield with nitrogen application. (**A**: Abd_FL, **B**: Abd_LF, **C**: Abd_SL **D**: Yield), The Abd_FL, Abd_LF, and Abd_SL are the abundances of flower, leaf, and soil, respectively, referring to the fraction of the given component within a pixel. The yield was obtained by drying and weighing and showed the mean yield of each nitrogen gradient.

In this paper, the abundance of flowers, leaves, and soils of each pixel was calculated by the fully constrained least-squares (FCLS) method. The ROI of each plot was selected to calculate its average abundance, and the relationship between abundance and nitrogen gradient was analyzed. The results are shown in **Figure 8**. With the increase of soil nitrogen application, rapeseed flower abundance decreased, but the overall change range was small, all approximately 0.2. The leaf abundance gradually increased from 0.2 to 0.7 with the augment of nitrogen application, showing an upward trend, and tended to be flat after N225. Soil abundance decreased gradually with the increase in nitrogen application rate. With the increase of nitrogen application, the yield of rapeseed increased gradually and reached the maximum value at N225. Nitrogen application continued to increase, but yield decreased. From **Figure 8**, it can be found that the changes in rapeseed leaf abundance and yield with nitrogen gradient have similar trends.

### Yield estimation using a combination of VIs and abundance data

In this study, the least squares regression analysis was performed, and leave−one−out cross−validation was conducted on images of different resolutions at UAV and satellite scales combined with flower and leaf abundance (Abd_FL_ and Abd_LF_). The results are shown in **Table 5**.

**Table 5:** The statistics of relationships of yield versus VI, VI × Abd_FL,_ and VI × Abd_LF_.

UAV	R^2^			RMSE (kg/ha)			CV (%)		
	VI	VI × Abd_FL_	VI × Abd_LF_	VI	VI × Abd_FL_	VI × Abd_LF_	VI	VI × Abd_FL_	VI × Abd_LF_
NDVI	0.50	0.09	0.84*	474.1	661.2	228.1*	20.6	28.6	9.9*
CIgreen	0.55	0.00	0.83*	409.5	685.6	249.7*	17.8	29.7	10.8*
VARI	0.18	0.01	0.79	609.0	697.3	273.4	26.4	30.2	11.8
RVI	0.51	0.02	0.84*	454.0	687.2	226.0*	19.7	29.8	9.8*
DVI	0.81	0.17	0.79	265.2	611.9	287.4	11.5	26.5	12.5
RDVI	0.81	0.01	0.82*	262.4	686.4	258.4*	11.4	29.7	11.2*
EVI	0.79	0.01	0.82*	284.7	692.1	260.1*	12.3	30.0	11.3*
TVI	0.81	0.15	0.80	263.0	619.3	284.2	11.4	26.8	12.3
NDYI	0.63	0.01	0.85*	406.8	686.5	216.2*	17.6	29.7	9.4*
GF-1	R^2^			RMSE (kg/ha)			CV (%)		
	VI	VI × Abd_FL_	VI × Abd_LF_	VI	VI × Abd_FL_	VI × Abd_LF_	VI	VI × Abd_FL_	VI × Abd_LF_
NDVI	0.01	0.44	0.36*	589.9	425.8	447.8*	20.1	13.1	14.1*
CIgreen	0.41	0.35	0.01	436.2	461.9	590.9	14.0	15.1	20.1
VARI	0.44	0.45	0.51*	425.7	420.1	399.5*	13.2	13.1	12.1*
RVI	0.01	0.46	0.30	585.8	422.1	468.8	20.2	13.0	15.6
DVI	0.48	0.47	0.48*	410.5	418.2	405.8*	12.6	12.5	12.6*
RDVI	0.39	0.46	0.44*	438.9	421.1	419.3*	13.3	12.8	13.1*
EVI	0.52	0.49	0.53*	398.6	407.7	389.4*	12.3	12.7	11.9*
TVI	0.49	0.48	0.50*	406.6	416.4	400.8*	12.2	12.6	12.3*
NDYI	0.06	0.11	0.03	563.7	526.5	606.6	20.4	18.5	20.1
Sentinel-2	R^2^			RMSE (kg/ha)			CV (%)		
	VI	VI × Abd_FL_	VI × Abd_LF_	VI	VI × Abd_FL_	VI × Abd_LF_	VI	VI × Abd_FL_	VI × Abd_LF_
NDVI	0.22	0.25	0.48*	429.4	411.8	329.3*	13.4	14.2	10.9*
CIgreen	0.14	0.44	0.29	443.9	345.3	394.5	13.9	12.2	11.8
VARI	0.24	0.03	0.41*	429.4	441.9	350.8*	14.0	14.5	11.4*
RVI	0.15	0.22	0.29	445.0	418.1	396.0	13.9	14.3	11.6
DVI	0.37	0.24	0.40*	375.5	412.4	367.7*	12.5	14.2	12.5*
RDVI	0.37	0.24	0.46*	368.1	411.5	346.3*	11.3	14.2	11.8*
EVI	0.43	0.18	0.45*	344.4	422.6	348.7*	11.4	14.2	11.9*
TVI	0.37	0.20	0.41*	374.2	418.8	366.6*	12.4	14.2	12.4*
NDYI	0.05	0.30	0.42	448.1	397.9	357.3	13.7	14.0	12.2

The top 6 performers were marked with * and bold, regardless of VI × Abd_FL_.

VIs, vegetation indices; NDVI, normalized difference vegetation index; CI_green_, green chlorophyll index; VARI, visible atmospherically resistant index; RVI, ratio vegetation index; DVI, difference vegetation index; RDVI, renormalized difference vegetation index; EVI, enhanced vegetation index; TVI, triangular vegetation index; NDYI, normalized difference yellowness index; UAV, unmanned aerial vehicle.

For the UAV scale, VI × Abd_FL_ reduced the correlation between VI and rapeseed yield (R^2^< 0.2), which was also confirmed by RMSE in **Table 5**. However, VI × Abd_LF_ did improve the correlation of the rapeseed yield regression model (R^2^ ≥ 0.79). For example, the R^2^ of the linear regression model using NDVI × Abd_LF_ inversion is more than 0.3 higher than the R^2^ inversion using NDVI alone. The R^2^ obtained with VARI × Abd_LF_ inversion is improved by more than 0.6. In addition, the CV was also significantly reduced (reduced by up to 11.8%).

For the satellite scale, VI × Abd_LF_ also had an overall improvement in R^2^ relative to VI except for CI_green_ from GF-1. VARI, RDVI, EVI, and TVI multiplied by Abd_LF_ from both GF-1 and Sentinel-2 had a medium correlation with statistical yield in **Table 4** (R^2^ > 0.4). At the same time, the values of RMSE decreased and have a certain reliability as well. NDVI and NDYI multiplied by Abd_LF_ from Sentinel-2 had a medium correlation with statistical yield. Compared with the relationship between VI and statistical yield, R^2^ has an increase of more than 0.2. However, this result was not evident in the results of GF-1.

**Figure 9:**
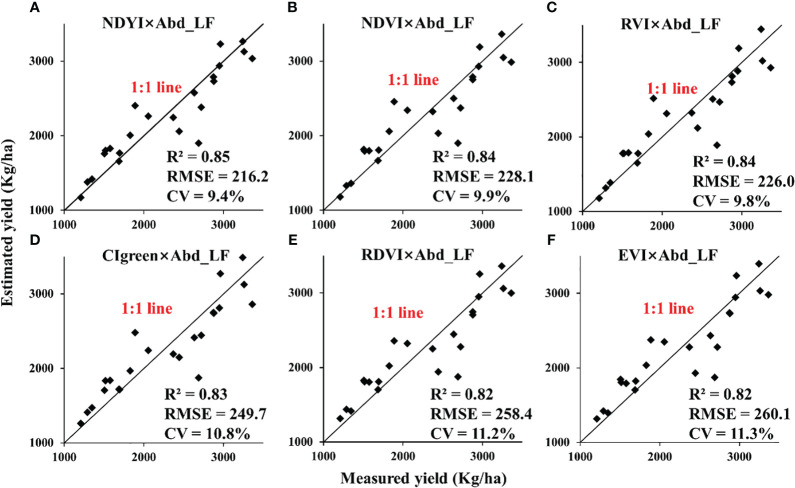
Validation for estimating rapeseed yield in 24 plots under different nitrogen treatments at the UAV scale. UAV, unmanned aerial vehicle. (**A**: NDYI×Abd_LF, **B**: NDVI×Abd_LF, **C**: RVI×Abd_LF, **D**: CIgreen×Abd_LF, **E**: RDVI×Abd_LF, **F**: EVI×Abd_LF).

**Figure 10:**
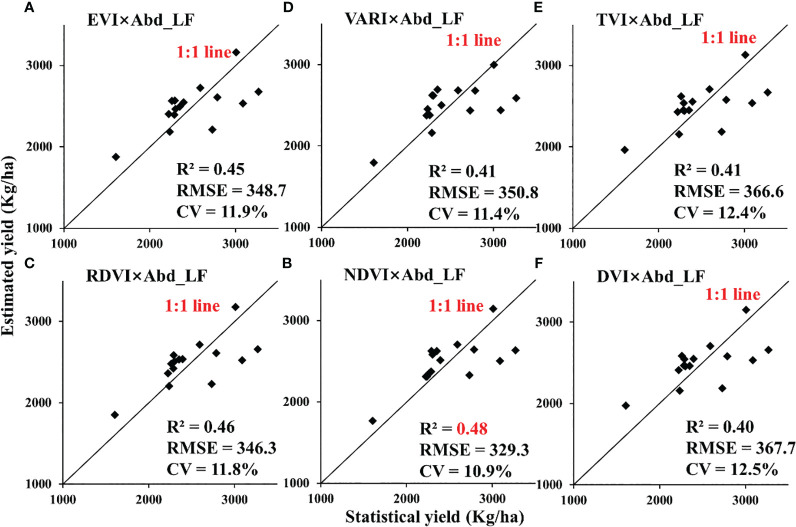
Validation for estimating rapeseed yield in Jianghan Plain at the satellite scale: Sentinel-2A/B in 2018. (**A**: EVI×Abd_LF, **B**: VARI×Abd_LF, **C**: TVI×Abd_LF, **D**: RDVI×Abd_LF, **E**: NDVI×Abd_LF, **F**: DVI×Abd_LF).

For different scales, retrieval models of VI × Abd_LF_ with higher R^2^ were selected as displayed in **Table 5** (the top 6 performers were marked with *, regardless of VI × Abd_FL_). They worked accurately in estimating the yield of rapeseed with RMSE below 260.1 kg/ha for the UAV scale and 447.8 kg/ha for the satellite scale (**Figures 9**, **10**). The CVs of estimated models with different scales were all less than 14.1%.

## Discussion

First of all, the purpose of this paper is not to study the method of rapeseed extraction, as rapeseed extraction is only a link to subsequent research. In this paper, a method that was easy to implement and suitable for China’s small field and fragmented planting mode was selected among the existing methods (Wang et al., 2018; Ashourloo et al., 2019; Zang et al., 2020; Zhang et al., 2022). Therefore, the CSRA method was adopted and obtained the results in **Figures 3**, **4** (Wang et al., 2018). Producer accuracy (PA) and user accuracy (UA) showed the feasibility of this method in rapeseed extraction. Since the spatial resolution of GF-1 WFV and Sentinel-2 MSI was 16 and 10, respectively, there were a large number of mixed pixels on the field boundary, which harmed the extraction accuracy and would lead to misclassification or omission of pixels. The flowering period of rapeseed was long and lasted for approximately 1 month. However, due to the difference in planting times and positions, the flowering proportions of rapeseed would differ, resulting in the missing of some pixels in the extraction results (D’ Andrimont et al., 2020). Pixel unmixing could improve pixel purity and alleviate problems caused by insufficient resolution (Somers et al., 2011).

At the flowering stage of rapeseed, NDYI performed better than NDVI in estimating yield at the UAV scale, which was consistent with Sulik and Long (2015). Numerous studies have demonstrated that NDVI has a good effect on yield at maximum chlorophyll content or maximum green canopy coverage (Lopes and Reynolds, 2012; Xiao et al., 2019; Gao et al., 2020). However, during the flowering period of rapeseed, the flowers affected the reflectance of leaves, increasing the reflectance of red and green bands, and the overall canopy was yellowish (**Figure 2**). NDVI did not perform as well in the case of reduced greenness. However, DVI, EVI, and TVI also had a relatively good linear relationship with the yield at flowering at both the UAV and the satellite scales (**Table 4**). In the flowering stage of rapeseed, the flowers were located above the canopy and were distributed in clusters. Flowers and leaves were significantly different in spectra (**Figure 2**). The reflectance of flowers is generally higher than that of leaves in the red and green bands and lower than that of leaves in the NIR band. With the increase in the flowering ratio, the effect of the flower on the canopy spectra also increased, resulting in an increase in the reflectance of the red band of the canopy. The values of NDVI and CI_green_ in the regions with high flowering proportions decreased, which may be the reason for the unsatisfactory yield estimation effect of NDVI, CI_green_, and RVI at the flowering stage of rapeseed.

For the UAV experiment in Wuxue City, different nitrogen applications affected the growth period of rapeseed (Li et al., 2016), which resulted in different proportions of flowering in the rapeseed at the same time (Fang et al., 2016). Plots with low nitrogen application entered the flowering stage earlier than those with normal nitrogen application, which could be the reason why the flower abundance of low nitrogen application in **Figure 8** was higher than that of high nitrogen application. The images used in this study might be missing some late-blooming flowers. Although the number of flowers is proportional to the number of rapeseed pods (Faraji, 2012), it was difficult to establish an accurate yield model from the one-phase images because of the difference in the flowering proportions. Rapeseed flower was not the main organ of photosynthesis, which made the use of flower abundance combined with VIs to estimate yield inaccurately (R^2^< 0.2). Under different nitrogen gradients, the canopy chlorophyll content and leaf area index of rapeseed had significant changes, which were closely related to the yield of rapeseed (Peng et al., 2019; Xu et al., 2020). Rapeseed leaf abundance represented the proportion of rapeseed leaves in a pixel, which avoided the influence of background spectra such as flowers and soil to a certain extent. In addition, leaf abundance and yield showed similar trends with nitrogen application (**Figure 8**). Therefore, the combination of VIs and rapeseed leaf abundance improved the accuracy of yield estimation. Our findings in the UAV experiment were consistent with the results obtained by GF-1 and Sentinel-2 (**Table 5**), which indicated that the combination of VIs and leaf abundance has certain applicability in yield estimation. Yield predictions can be made over a wide range, especially for vegetation such as rapeseed with distinct spectral differences between flowers and leaves.

When using satellite images to estimate yield at the flowering stage of rapeseed, NDVI and NDYI cannot reach the accuracy of R^2^ above 0.66 as John J. Sulik mentioned in the North Dakota experiment (Sagan et al., 2021), which could be related to different planting patterns. The United States mainly plants on large farms on a large scale, with flat terrain and a single crop. However, China’s terrain is complex, and the fields are scattered and broken (**Figure 4**). In the analysis of satellite images, the phenomenon of mixed pixels was serious, so the field boundary and soil harm the reflectance of rapeseed. At the same time, flowering time was also affected by sowing time, rainfall, degree of flowering, and soil fertility. Therefore, according to the characteristics of China’s fields, the method of using VIs to retrieve crop yield at the satellite scale is limited to a certain extent. We had to consider using SMA techniques to improve the yield estimation models. Compared with the VI models, R^2^ still had a certain improvement in the improved models with leaf abundance at the satellite scale though not much of an increase (the increment of R^2^ was less than 0.1 mostly; **Table 5**). Some scholars have proposed that the abundance of endmembers has a spatial correlation (Jia and Qian, 2007), which means that abundance is not simply independent of each other. With the decrease in spatial resolution, the mixed pixel phenomenon becomes more obvious, and the extraction accuracy of abundance also decreases. Therefore, compared with the VI model, the accuracy of the VI × Abd_LF_ model in satellite scale yield estimation was improved, but the improvement was not significant enough. In conclusion, considering the scale of UAV and satellite, the experimental results showed that considering SMA could improve the limitation of using only VIs to retrieve crop yield.

The theoretical framework provided in this study is applicable to the flowering period of rapeseed, especially when there is a significant difference between flowers and leaves during the flowering period of vegetation. Follow-up attempts will be made to research other crops. For large-scale yield estimation, this study can establish a better yield estimation model in the plain area only through a single temporal image of rapeseed during flowering. In the future, we will consider more factors such as terrain, weather, rainfall, phenology, and other SMAs to enhance the robustness of the model.

## Conclusions

**Figure 11:**
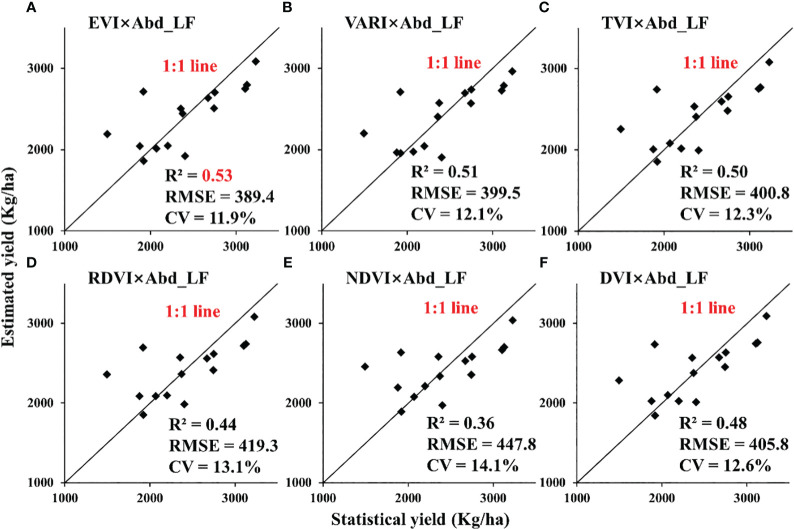
Validation for estimating rapeseed yield in Jianghan Plain at the satellite scale: GF-1 in 2014. (**A**: EVI×Abd_LF, **B**: VARI×Abd_LF, **C**: TVI×Abd_LF, **D**: RDVI×Abd_LF, **E**: NDVI×Abd_LF, **F**: DVI×Abd_LF).

In this study, a method for retrieving rapeseed yield using leaf abundance multiplied by VIs was adopted and compared at the UAV and satellite scales. We found that there was a prominent spectral difference between flower and leaf during the flowering period of rapeseed, which often appeared in the image in the form of mixed pixels. Therefore, we tried to extract the abundance of different components of rapeseed by SMA technology and estimated rapeseed yield by combining VIs. The product of VIs selected in this paper and leaf abundance was closely related to rapeseed yield for the UAV-scale nitrogen gradient experiment, which was better than the simple VI model for yield estimation, with R^2^ above 0.78 (**Figure 9**). The yield estimation models of NDVI × Abd_LF_, RVI × Abd_LF_, and NDYI × Abd_LF_ had the highest accuracy, and the CVs were below 10%. For the satellite scale, most of the estimation models of the product of VIs and rapeseed leaf abundance were also improved compared with the simple VI model, with R^2^ above 0.4 (**Figures 11**, **10**). Among them, RDVI × Abd_LF_ and EVI × Abd_LF_ had a steady improvement, with CVs below 13.1%. Moreover, the yield estimation models of NDVI × Abd_LF_, VARI × Abd_LF_, RDVI × Abd_LF_, and EVI × Abd_LF_ had consistent performances at both UAV and satellite scales. The experimental results showed that considering SMA could improve the limitation of using only VIs to retrieve rapeseed yield at the flowering stage.

## Data availability statement

The raw data supporting the conclusions of this article will be made available by the authors, without undue reservation.

## Author contributions

All authors have made significant contributions to this research. YG conceived the research ideas. SF and YP designed the experiments. YL conducted the data analysis and provided the writing of this paper. NY and SL performed the majority of the data processing, and KY provided data to back it up. All authors contributed to the article and approved the submitted version.

## References

[B1] Ashourloo D. Shahrabi H. S. Azadbakht M. Aghighi H. Nematollahi H.. (2019). Automatic canola mapping using time series of sentinel 2 images. Isprs J. Photogrammetry Remote Sens. 156, 63–76. doi: 10.1016/j.isprsjprs.2019.08.007

[B2] Atzberger C. (2013). Advances in remote sensing of agriculture: context description, existing operational monitoring systems and major information needs. Remote Sens. 5, 949–981. doi: 10.3390/rs5020949

[B3] Baugh W. M. Groeneveld D. P. (2008). Empirical proof of the empirical line. Int. J. Remote Sens. 29, 665–672. doi: 10.1080/01431160701352162

[B4] Behrens T. Müller J. Diepenbrock W. (2006). Utilization of canopy reflectance to predict properties of oilseed rape (brassica napus l.) And barley (hordeum vulgare l.) During ontogenesis. Eur. J. Agron. 25, 345–355. doi: 10.1016/j.eja.2006.06.010

[B5] Broge N. H. Leblanc E. (2001). Comparing prediction power and stability of broadband and hyperspectral vegetation indices for estimation of green leaf area index and canopy chlorophyll density. Remote Sens. Environ. 76, 156–172. doi: 10.1016/S0034-4257(00)00197-8

[B6] D’ Andrimont R. Taymans M. Lemoine G. Ceglar A. Yordanov M.. (2020). Detecting flowering phenology in oil seed rape parcels with sentinel-1 and -2 time series. Remote Sens. Environ. 239, 111660. doi: 10.1016/j.rse.2020.111660 32184531 PMC7043338

[B7] Duan B. Fang S. Zhu R. Wu X. Wang S.. (2019). Remote estimation of rice yield with unmanned aerial vehicle (uav) data and spectral mixture analysis. Front. Plant Sci. 10. doi: 10.3389/fpls.2019.00204 PMC6400984 30873194

[B8] Dwyer J. L. Kruse F. A. Lefkoff A. B. (1995). Effects of empirical versus model-based reflectance calibration on automated analysis of imaging spectrometer data; A case study from the drum mountains, utah. Photogrammetric Eng. Remote Sens. 61, 1247–1254.

[B9] Fang S. Tang W. Peng Y. Gong Y. Dai C.. (2016). Remote estimation of vegetation fraction and flower fraction in oilseed rape with unmanned aerial vehicle data. Remote Sens. 8, 416. doi: 10.3390/rs8050416

[B10] Faraji A. (2012). Flower formation and pod/flower ratio in canola (brassica napus l.) Affected by assimilates supply around flowering. Int. J. Plant Production 4, 271–280. doi: 10.22069/ijpp.2012.710

[B11] Fielding A. H. Bell J. F. (1997). A review of methods for the assessment of prediction errors in conservation presence/absence models. Environ. Conserv. 24, 38–49. doi: 10.1017/S0376892997000088

[B12] Gao L. Wang X. Johnson B. A. Tian Q. Wang Y.. (2020). Remote sensing algorithms for estimation of fractional vegetation cover using pure vegetation index values: a review. Isprs J. Photogrammetry Remote Sens. 159, 364–377. doi: 10.1016/j.isprsjprs.2019.11.018 PMC7613353 36082112

[B13] García-Martínez H. Flores-Magdaleno H. Ascencio-Hernández R. Khalil-Gardezi A. Tijerina-Chávez L.. (2020). Corn grain yield estimation from vegetation indices, canopy cover, plant density, and a neural network using multispectral and rgb images acquired with unmanned aerial vehicles. Agriculture 10, 277. doi: 10.3390/agriculture10070277

[B14] Gitelson A. A. (2005). Remote estimation of canopy chlorophyll content in crops. Geophysical Res. Lett. 32, 1–4. doi: 10.1029/2005GL022688

[B15] Gitelson A. A. Kaufman Y. J. Stark R. Rundquist D. (2002). Novel algorithms for remote estimation of vegetation fraction. Remote Sens. Environ. 80, 76–87. doi: 10.1016/S0034-4257(01)00289-9

[B16] Guan K. Wu J. Kimball J. S. Anderson M. C. Frolking S.. (2017). The shared and unique values of optical, fluorescence, thermal and microwave satellite data for estimating large-scale crop yields. Remote Sens. Environ. 199, 333–349. doi: 10.1016/j.rse.2017.06.043

[B17] Heinz D. Chang C. Althouse M. L. (1999). “"Fully constrained least-squares based linear unmixing [hyperspectral image classification]",” in IEEE 1999 International Geoscience and Remote Sensing Symposium. IGARSS'99 (Cat. No. 99CH36293) (IEEE). doi: 10.1109/IGARSS.1999.774644

[B18] Jia S. Qian Y. (2007). Spectral and spatial complexity-based hyperspectral unmixing. IEEE Trans. On Geosci. Remote Sens. 45, 3867–3879. doi: 10.1109/TGRS.2007.898443

[B19] Jordan C. F. (1969). Derivation of leaf-area index from quality of light on the forest floor. Ecology 50, 663–666. doi: 10.2307/1936256

[B20] Kira O. Linker R. Gitelson A. (2015). Non-destructive estimation of foliar chlorophyll and carotenoid contents: focus on informative spectral bands. Int. J. Appl. Earth Observation Geoinformation 38, 251–260. doi: 10.1016/j.jag.2015.01.003

[B21] Laliberte A. S. Goforth M. A. Steele C. M. Rango A. (2011). Multispectral remote sensing from unmanned aircraft: image processing workflows and applications for rangeland environments. Remote Sens. 3, 2529–2551. doi: 10.3390/rs3112529

[B22] Li X. Li Q. Yang T. Nie Z. Chen G.. (2016). Responses of plant development, biomass and seed production of direct sown oilseed rape (brassica napus) to nitrogen application at different stages in yangtze river basin. Field Crops Res. 194, 12–20. doi: 10.1016/j.fcr.2016.04.024

[B23] Liao C. Wang J. Dong T. Shang J. Liu J.. (2019). Using spatio-temporal fusion of landsat-8 and modis data to derive phenology, biomass and yield estimates for corn and soybean. Sci. Total Environ. 650, 1707–1721. doi: 10.1016/j.scitotenv.2018.09.308 30273730

[B24] Liu H. Q. Huete A. (1995). A feedback based modification of the ndvi to minimize canopy background and atmospheric noise. IEEE Trans. On Geosci. Remote Sens. 33, 457–465. doi: 10.1109/TGRS.1995.8746027

[B25] Lobell D. B. Cassman K. G. Field C. B. (2009). Crop yield gaps: their importance, magnitudes, and causes. Annu. Rev. Environ. Resour. 34, 179–204. doi: 10.1146/annurev.environ.041008.093740

[B26] Lopes M. S. Reynolds M. P. (2012). Stay-green in spring wheat can be determined by spectral reflectance measurements (normalized difference vegetation index) independently from phenology. J. Exp. Bot. 63, 3789–3798. doi: 10.1093/jxb/ers071 22412185 PMC3388823

[B27] Ma N. Yuan J. Li M. Li J. Zhang L.. (2014). Ideotype population exploration: growth, photosynthesis, and yield components at different planting densities in winter oilseed rape (brassica napus l.). PloS One 9, e114232. doi: 10.1371/journal.pone.0114232 25517990 PMC4269386

[B28] Maes W. H. Steppe K. (2019). Perspectives for remote sensing with unmanned aerial vehicles in precision agriculture. Trends Plant Sci. 24, 152–164. doi: 10.1016/j.tplants.2018.11.007 30558964

[B29] Maimaitijiang M. Sagan V. Sidike P. Hartling S. Esposito F.. (2020). Soybean yield prediction from uav using multimodal data fusion and deep learning. Remote Sens. Environ. 237, 111599. doi: 10.1016/j.rse.2019.111599

[B30] Mulla D. J. (2013). Twenty five years of remote sensing in precision agriculture: key advances and remaining knowledge gaps. Biosyst. Eng. 114, 358–371. doi: 10.1016/j.biosystemseng.2012.08.009

[B31] Munghemezulu C. Combrinck L. Botai J. O. Chirima G. Mashaba Z.. (2017). Forecasting winter wheat yields using modis ndvi data for the central free state region. South Afr. J. Sci. 113, 1–6. doi: 10.17159/sajs.2017/20160201

[B32] Nagy A. Fehér J. Tamás J. (2018). Wheat and maize yield forecasting for the tisza river catchment using modis ndvi time series and reported crop statistics. Comput. Electron. Agric. 151, 41–49. doi: 10.1016/j.compag.2018.05.035

[B33] Pan B. Shi Z. An Z. Jiang Z. Ma Y. (2017). A novel spectral-unmixing-based green algae area estimation method for goci data. IEEE J. Selected Topics Appl. Earth Observations Remote Sens. 10, 437–449. doi: 10.1109/JSTARS.2016.2585161

[B34] Peng Y. Zhu T. E. Li Y. Dai C. Fang S.. (2019). Remote prediction of yield based on lai estimation in oilseed rape under different planting methods and nitrogen fertilizer applications. Agric. For. Meteorology 271, 116–125. doi: 10.1016/j.agrformet.2019.02.032

[B35] Pu H. Chen Z. Wang B. Xia W. (2015). Constrained least squares algorithms for nonlinear unmixing of hyperspectral imagery. IEEE Trans. On Geosci. Remote Sens. 53, 1287–1303. doi: 10.1109/TGRS.2014.2336858

[B36] Ray S. S. Jain N. Miglani A. Singh J. P. Singh A. K.. (2010). Defining optimum spectral narrow bands and bandwidths for agricultural applications. Curr. Sci. (Bangalore) 98, 1365–1369.

[B37] Ren T. Zou J. Wang Y. Li X. Cong R. (2016). Estimating nutrient requirements for winter oilseed rape based on quefts analysis. J. Agric. Sci. 154, 425–437. doi: 10.1017/S0021859615000301

[B38] Richardson A. J. Wiegand C. L. (1977). Distinguishing vegetation from soil background information. Photogrammetric Eng. Remote Sens. 12, 1541–1552.

[B39] Roujean J. Breon F. (1995). Estimating par absorbed by vegetation from bidirectional reflectance measurements. Remote Sens. Environ. 51, 375–384. doi: 10.1016/0034-4257(94)00114-3

[B40] Rouse J. W. Haas R. H. Schell J. A. Deering D. W. (1974). Monitoring vegetation systems in the great plains with erts. Nasa Spec. Publ. 351, 309. Available at: https://ntrs.nasa.gov/citations/19740022614

[B41] Sagan V. Maimaitijiang M. Bhadra S. Maimaitiyiming M. Brown D. R.. (2021). Field-scale crop yield prediction using multi-temporal worldview-3 and planetscope satellite data and deep learning. Isprs J. Photogrammetry Remote Sens. 174, 265–281. doi: 10.1016/j.isprsjprs.2021.02.008

[B42] Sakamoto T. Gitelson A. A. Arkebauer T. J. (2014). Near real-time prediction of u.s. Corn yields based on time-series modis data. Remote Sens. Environ. 147, 219–231. doi: 10.1016/j.rse.2014.03.008

[B43] Segarra J. Araus J. L. Kefauver S. C. (2022). Farming and earth observation: sentinel-2 data to estimate within-field wheat grain yield. Int. J. Appl. Earth Observation Geoinformation 107, 102697. doi: 10.1016/j.jag.2022.102697

[B44] Singer R. B. McCord T. B. Merrill R. B. Bogard D. D. Hoerz F.. (1979). Mars; Large scale mixing of bright and dark surface materials and implications for analysis of spectral reflectance. Proc. Lunar Planetary Sci. Conf. 2, 1835–1848.

[B45] Somers B. Asner G. P. Tits L. Coppin P. (2011). Endmember variability in spectral mixture analysis: a review. Remote Sens. Environ. 115, 1603–1616. doi: 10.1016/j.rse.2011.03.003

[B46] Son N. T. Chen C. F. Chen C. R. Minh V. Q. Trung N. H. (2014). A comparative analysis of multitemporal modis evi and ndvi data for large-scale rice yield estimation. Agric. For. Meteorology 197, 52–64. doi: 10.1016/j.agrformet.2014.06.007

[B47] Sulik J. J. Long D. S. (2015). Spectral indices for yellow canola flowers. Int. J. Remote Sens. 36, 2751–2765. doi: 10.1080/01431161.2015.1047994

[B48] Toth C. Jóźków G. (2016). Remote sensing platforms and sensors: a survey. Isprs J. Photogrammetry Remote Sens. 115, 22–36. doi: 10.1016/j.isprsjprs.2015.10.004

[B49] Tucker C. J. (1979). Red and photographic infrared linear combinations for monitoring vegetation. Remote Sens. Environ. 8, 127–150. doi: 10.1016/0034-4257(79)90013-0

[B50] Wang D. Fang S. Yang Z. Wang L. Tang W.. (2018). A regional mapping method for oilseed rape based on hsv transformation and spectral features. Isprs Int. J. Geo-Information 7, 224. doi: 10.3390/ijgi7060224

[B51] Xiao J. Chevallier F. Gomez C. Guanter L. Hicke J. A.. (2019). Remote sensing of the terrestrial carbon cycle: a review of advances over 50 years. Remote Sens. Environ. 233, 111383. doi: 10.1016/j.rse.2019.111383

[B52] Xu H. Cen H. Ma Z. Wan L. Zhou W.. (2020). Assessment of seed yield and quality of winter oilseed rape using chlorophyll fluorescence parameters of pods. Trans. Asabe 63, 231–242. doi: 10.13031/trans.13176

[B53] Yu N. Li L. Schmitz N. Tian L. F. Greenberg J. A.. (2016). Development of methods to improve soybean yield estimation and predict plant maturity with an unmanned aerial vehicle based platform. Remote Sens. Environ. 187, 91–101. doi: 10.1016/j.rse.2016.10.005

[B54] Zang Y. Chen X. Chen J. Tian Y. Shi Y.. (2020). Remote sensing index for mapping canola flowers using modis data. Remote Sens. 12, 3912. doi: 10.3390/rs12233912

[B55] Zhang H. Liu W. Zhang L. (2022). Seamless and automated rapeseed mapping for large cloudy regions using time-series optical satellite imagery. Isprs J. Photogrammetry Remote Sens. 184, 45–62. doi: 10.1016/j.isprsjprs.2021.12.001

[B56] Zhang Y. Wan Y. Wang B. Kang Y. Xiong J. (2015). Automatic processing of chinese gf-1 wide field of view images. Int. Arch. Photogramm. Remote Sens. Spatial Inf. Sci. XL-7/W3. 729–734. doi: 10.5194/isprsarchives-XL-7-W3-729-2015

[B57] Zhou X. Zheng H. B. Xu X. Q. He J. Y. Ge X. K.. (2017). Predicting grain yield in rice using multi-temporal vegetation indices from uav-based multispectral and digital imagery. Isprs J. Photogrammetry Remote Sens. 130, 246–255. doi: 10.1016/j.isprsjprs.2017.05.003

